# The Effect of Chronic and Inhospital Exposure to Renin-Angiotensin System Inhibitors on the Outcome and Inflammatory State of Coronavirus Disease 2019 Adult Inpatients

**DOI:** 10.1155/2021/5517441

**Published:** 2021-03-08

**Authors:** Pedro Gaspar, Inês Parreira, Pedro Antunes Meireles, Filipe Bessa, Virgílio Dias Silva, Ana Mafalda Abrantes, António Pais de Lacerda, Catarina Mota

**Affiliations:** ^1^Serviço de Medicina 2, Hospital de Santa Maria, Centro Hospitalar Universitário Lisboa Norte, Lisbon, Portugal; ^2^Faculdade de Medicina, Universidade de Lisboa, Lisbon, Portugal; ^3^Serviço de Oncologia Médica, Instituto Português de Oncologia de Lisboa Francisco Gentil, Lisbon, Portugal; ^4^Serviço de Medicina Intensiva, Hospital de Santa Maria, Centro Hospitalar Universitário Lisboa Norte, Lisbon, Portugal; ^5^Instituto de Medicina Molecular, João Lobo Antunes, Faculdade de Medicina, Universidade de Lisboa, Lisbon, Portugal

## Abstract

**Background:**

Controversies exist about the effect of renin-angiotensin system inhibitors (RASi) on coronavirus disease 2019 (COVID-19) outcome. The inhospital use of RASi and its effect on inflammatory sate are still poorly studied during the COVID-19 pandemic.

**Objectives:**

We aimed to compare the impact of previous and inhospital RASi exposure on the outcome and inflammatory response of COVID-19 patients.

**Methods:**

Single-centre, ambispective analysis of hospitalized adult COVID-19 patients at Hospital de Santa Maria, Lisbon, between March and August 2020 was performed. We excluded asymptomatic patients and those admitted due to another disease. The primary outcome was inhospital all-cause mortality. Illness severity was assessed based on the development of acute respiratory distress syndrome/acute lung injury (ARDS/ALI), intensive care unit (ICU) admission, and need for invasive mechanical ventilation (IMV). We used C-reactive protein (CRP), ferritin, and interleukin 6 (IL-6) as surrogate markers of the inflammatory response.

**Results:**

From a total of 432 patients, 279 were selected, among whom 133 (47.7%) were receiving a RASi. Chronic treatment with RASi was not associated with the risk of death (OR 1.24, 95% CI 0.66–2.31, *p*=0.500), ARDS/ALI development (OR 1.12, 95% CI 0.67–1.86, *p*=0.676), ICU admission (OR 1.11, 95% CI 0.67–1.84, *p* = 0.686), and IMV need (OR 1.03, 95% CI 0.58–1.84, *p*=0.917) in a univariable and multivariable analysis. Inhospital RASi withdrawing was associated with the risk of death (OR 4.38, 95% CI 1.11–17.21, *p*=0.035) and ARDS/ALI development (OR 4.33, 95% CI 1.49–12.6, *p*=0.007), the latter remaining significant after adjustment. Previous exposure to RASi was associated with lower CRP levels at admission (*p*=0.018). IL-6 levels were significantly higher in those patients whose RASi were stopped (*p*=0.024).

**Conclusion:**

Previous and inhospital exposure to RASi was not associated with mortality nor severity of COVID-19. This study supports current guidance on RASi management during the COVID-19 pandemic.

## 1. Introduction

The coronavirus disease 2019 (COVID-19) has caused more than two and a half million deaths around the globe. Despite the experimental treatments that have been tried, the pandemic is still evolving, so every effort must be made to reduce its morbidity and mortality.

The renin-angiotensin system inhibitors (RASi), namely, angiotensin-converting enzyme inhibitor (ACEi) and angiotensin II receptor type 1 blocker (ARB), are widely used drugs and represent first-line therapies for the treatment for high blood pressure (HBP) [1], a common comorbid condition in COVID-19 patients and a known risk factor for inhospital death [2]. The discovery that the COVID-19 pathogen, the severe acute respiratory syndrome new coronavirus 2 (SARS-CoV-2), uses angiotensin-converting enzyme 2 (ACE2) for viral entry [3] sparked a lively discussion about whether RASi exposure could modulate the clinical course of COVID-19.

The renin-angiotensin system (RAS) relies on the balance between two opposite arms. On one side, angiotensin (Ang) II exerts vasoconstrictive, pro-proliferative, and proinflammatory effects via Ang II receptor type 1. On the other side, Ang 1–7, produced from the cleavage of Ang II by ACE2, has opposite actions [4]. Preclinical models showed that chronic exposure to RASi increased tissue ACE2 expression [5, 6]. Conversely, ACE2 is significantly downregulated after SARS-CoV-2 binding [3], creating an imbalance between the two RAS axes towards Ang II effects.

The acute respiratory distress syndrome (ARDS) is the main cause of death in COVID-19 patients [7]. Its pathophysiology seems to depend at least in part on the RAS instability. Severe ARDS secondary to impaired ACE2 activity has been identified in other viral pneumonias, like H7N9 influenza and SARS-CoV, where elevated circulating Ang II was associated with disease progression and higher mortality rates [8, 9]. In a group of 35 ARDS patients, concentrations of Ang 1–7 were higher in patients who survived, suggesting a higher ACE2 activity in such patients [10]. In line with this, serum levels of Ang II were also significantly elevated in COVID-19 patients and exhibited a linear positive correlation with viral load and lung injury [11].

Despite that the safety of RASi use during the pandemic has been assured, data shows conflicting evidence regarding its effect on disease severity, possibly due to a lack of standardized definitions [12]. The growing body of evidence on this topic comes from observational retrospective studies and, as far as we know, no prospective analysis has been published. In addition, most of the data come from Asian countries, mainly from China. There is a lack of data from European countries and, in particular, from Portugal, where no similar study has been published so far.

In the present study, we aim to investigate the effect of chronic RASi exposure on inhospital mortality and clinical severity of COVID-19 as measured by the development of ARDS/acute lung injury (ALI), intensive care unit (ICU) admission, and the requirement for invasive mechanical ventilation (IMV). We also sought to explore how RASi inhospital management influences those clinical outcomes and the inflammatory response.

## 2. Materials and Methods

### 2.1. Study Design and Participants

**Figure 1:**
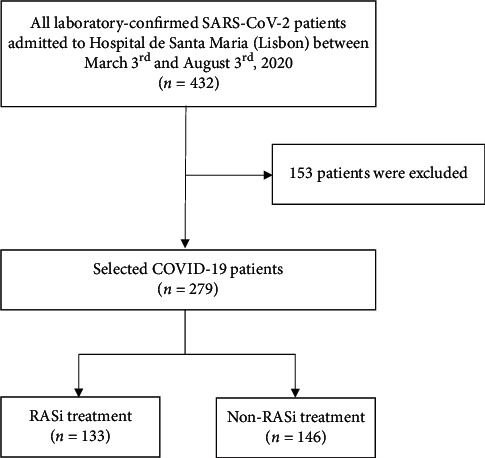
Patients' selection process. COVID-19, coronavirus disease 2019; RASi, renin-angiotensin system inhibitors; SARS-CoV-2, severe acute respiratory syndrome coronavirus 2.

This is an ambispective open cohort study of all adult COVID-19 patients admitted to Hospital de Santa Maria (Lisbon, Portugal), between March 3^rd^ and August 3^rd^, 2020. The clinical outcomes were recorded up until September 3^rd^, 2020. All patients had a positive test for severe acute respiratory syndrome coronavirus 2 (SARS-CoV-2) by reverse-transcriptase polymerase chain reaction (RT-PCR). We have excluded all patients that were hospitalized due to another disease (despite having tested positive for SARS-CoV-2 by RT-PCR), those admitted due to social/sanitary reasons only (e.g., inability of social isolation), and pregnant women. The patients' selection process is schematized in Figure 1. Patients entered the study retrospectively and prospectively before and after the 27^th^ of April, respectively. Patients were followed until they died, were lost to follow-up, or were discharged. All patients reached the end of the study. Patients were stratified according to RASi exposure: RASi group vs. non-RASi group. Patients were classified in the RASi group as long as they were on any ACEi/ARB upon admission, despite the dose and duration of treatment and regardless of how RASi therapy was subsequently managed during the inhospital stay. For a subanalysis purpose, we then focused on how the RASi treatment was managed during the first three days of hospitalization. We have looked into whether patients had begun, stopped, or continued the RASi treatment regimen and categorized them into three groups, start-RASi group, stop-RASi group, and non-stop-RASi group, accordingly.

The study was approved by the ethics committee of Hospital de Santa Maria (N°177/20) and complies with the Declaration of Helsinki statement.

### 2.2. Data Collection

Patients' demographic characteristics, comorbid conditions, and clinical manifestations were obtained through clinical interviews and the analysis of individual patient electronic files. Laboratory results were collected using electronic software. Comorbidities were either self-reported by the patients and/or extracted from their medical records. Patients' treatment regimens were assessed through medical interviews and confirmed in the prescribed medication chart. The inhospital treatments were collected from electronic files and prescription software. All data were collected by four physicians and reviewed by other two. The anonymity of the collected data was warranted by the authors, and data were collected and stored according to the applicable legislation.

### 2.3. Definitions and Outcomes

The primary outcome was inhospital all-cause mortality. The COVID-19 severity was assessed according to the secondary outcomes as follows: development of ARDS/ALI, ICU admission, and need for IMV. ARDS and ALI were defined according to Berlin criteria [13] and the American-European Consensus Conference on ARDS [14], respectively. The COVID-19 onset was defined as the time point when symptoms were first noticed. Symptoms were considered up until the third day of hospitalization. Laboratory results corresponded to the first available result up to the third day of inhospital stay. We considered the outcome date as the time the patient died or was clinically discharged (no fever and no supplemental oxygen requirements for more than two consecutive days). The total length of hospital stay was determined from the date of admission until the outcome date.

### 2.4. Statistical Approach

Statistical analyses were conducted using STATA® software (version 16) and SPSS® (version 26.0). Continuous variables were expressed as median ± interquartile range and categorical variables as number (%). We compared groups of patients using Pearson's chi-square test and Fisher exact test for categorical variables and the Mann–Whitney *U* test for continuous numerical variables. Odds ratios and multivariable analysis were performed using a logistic model. For multivariable analysis, we included variables from the univariable analysis if their between-group differences were significant. Patients were censored if they were lost to follow-up or reached the primary outcome. The significance level was defined at 0.05.

## 3. Results

### 3.1. Demographics, Comorbidities, and Disease Characteristics

From a total of 432 patients admitted during the study period time, 279 patients were selected for analysis. The retrospective and prospective parts of the study included 117 (41.9%) and 162 patients (58.1%), respectively. One hundred thirty-three patients (47.7%) belonged to the RASi group, whereas 146 patients (52.3%) were categorized in the non-RASi group. The baseline demographic, clinical, and laboratory characteristics as well as inpatient treatments and outcomes of our cohort are summarized in Table 1. Patients in the RASi group had a comparable gender distribution (male, 57.9% vs. 56.2%, *p*=0.771) but older age (76 ± 21 vs. 64 ± 30 years, *p* < 0.001) and higher frequency of Caucasian ethnicity (88.3% vs. 78.0%, *p*=0.026) than the patients in the non-RASi group. Among all patients, HBP was the most frequent comorbid condition (66.3%), followed by dyslipidaemia (33.3%), diabetes mellitus (DM) (27.2%), cardiovascular disease (CVD) (26.5%), obesity (25.5%), and chronic kidney disease (CKD) (18.4%). Compared to patients in the non-RASi group, we observed a higher prevalence of HBP (39.7% vs. 95.5%, *p* < 0.001), dyslipidaemia (21.2% vs. 46.6%, *p* < 0.001), DM (15.8% vs. 39.9%, *p* < 0.001), CVD (17.8% vs. 36.1%, *p*=0.001), obesity (19.2% vs. 32.6%, *p*=0.011), and CKD (13.8% vs. 23.3%, *p*=0.041) in the RASi group patients. Cerebrovascular disease, chronic obstructive pulmonary disease (COPD), and active cancer were similarly distributed between both groups.

Both groups showed similar signs and symptoms except for cough (74.5% vs. 58.8, *p*=0.006), headache (21% vs. 7.7%, *p*=0.002), ageusia (11.3% vs. 3.9%, *p*=0.024), and anosmia (9.2% vs. 3.1%, *p*=0.046) that were significantly more prevalent in the non-RASi group than in the RASi group. Patients under RASi therapy presented sooner to the hospital, with a significantly smaller median time from symptom onset to hospital admission (4 ± 4 vs. 6 ± 5 days, *p*=0.041). The median duration of total hospital stay was similar between the two groups (11 ± 13 vs. 10 ± 10.5 days, *p*=0.669) (Table 1).

### 3.2. Association between RASi Exposure with Mortality and Severe Clinical Outcomes

Forty-eight patients (17.3%) died during the study period, and two were transferred to another hospital during ICU stay, hence lost to follow-up. Patients who died were older (84.5 ± 16.5 vs. 67 ± 25 years, *p* < 0.001) and presented sooner to the hospital (3 ± 4 vs. 6 ± 4 days, *p*=0.008). CVD (50.0% vs. 21.8%, *p* < 0.001), COPD (29.2% vs. 10.0%, *p* < 0.001), HBP (83.3% vs. 62.9%, *p*=0.006), and CKD (31.3% vs. 15.8%, *p*=0.012) were significantly more prevalent in COVID-19 patients who died compared to those who survived. Supplementary Table 1 complements the demographic and clinical characterization of survivors vs. nonsurvivors.

Eighty-seven patients (87/269, 32.3%) developed ARDS/ALI, 89 patients (89/279, 31.9%) were admitted to the ICU, and 58 patients (58/279, 20.8%) needed IMV. Patients who reached these outcomes were more frequently of male gender (*p*=0.001, *p* < 0.001, and *p*=0.001, respectively), whereas there was no difference in age or in ethnic origin. In general, the proportion of comorbid conditions were similarly distributed between patients who reached any of these severe clinical outcomes compared to those who had a less severe clinical course, except for COPD which was significantly more prevalent in patients who were admitted to ICU than in those who were not (20.2% vs. 10.0%, *p* = 0.019). See Supplementary Tables 2–4 for further characterization.

Regarding the use of RASi, we observed no difference in inhospital mortality rate (18.9% vs. 15.9%, *p*=0.499), development of ARDS/ALI (33.6% vs. 31.2%, *p*=0.676), ICU admission (33.1% vs. 30.8%, *p*=0.686), and need for IMV (21.1% vs. 20.6%, *p*=0.917) between patients on the RASi group and those in the non-RASi group, despite a uniformly greater percentage in the former group. In a logistic model, the association between RASi use and any of the aforementioned outcomes remained without statistical significance after adjusting for confounder variables (Table 2). Additionally, we noticed that patients in the RASi group had shorter time from disease onset to ARDS/ALI (7 ± 5 vs. 11 ± 6 days, *p*=0.002), to ICU admission (7 ± 5 v*s*. 8 ± 3.5 days, *p*=0.044), and to IMV (6 ± 5 vs. 9 ± 4 days, *p*=0.002) than in those in the non-RASi group. Time from symptom onset to death was similar between the two groups (*p*=0.732) (Table 1).

### 3.3. Effect of Maintenance vs. Suspension of RASi during Inhospital Stay

Focusing on the RASi group (*n* = 133), we compared primary and secondary outcomes between patients who stopped and those who maintained RASi on hospital admission. This information was available in 88 patients (88/133, 66.2%). Supplementary Table 5 summarizes the demographic and clinical characteristics and inpatient outcome of the two groups. Patients whose RASi were stopped were older (80 ± 16 vs. 71 ± 24 years, *p*=0.033) and were more frequently of Caucasian ethnicity (90.0% vs. 80.0%, *p*=0.045) than patients whose RASi therapy was not stopped. Both groups were comparable in gender distribution and comorbid conditions. In univariable analysis, patients in the stop-RASi group showed a higher risk of dying (unadjusted odds ratio (OR) 4.38, 95% CI 1.11–17.21, *p*=0.035) and of developing ARDS/ALI (unadjusted OR 4.33, 95% CI 1.49–12.59, *p*=0.007). The risk of ICU admission (OR 2.00, 95% CI 0.73–5.47, *p*=0.175) and need for IMV (OR 2.27, 95% CI 0.53–9.72, *p*=0.269) were similar between the two groups (Table 3). Only the association between RASi withdrawing and ARDS/ALI development remained statistically significant after adjustment (adjusted OR 4.54, 95% CI 1.53–13.44, *p*=0.006).

### 3.4. Effect of Previous RASi Exposure and Inhospital Withdrawing on Inflammatory Markers

We used the levels of C-reactive protein (CRP), ferritin, and interleukin 6 (IL-6) as surrogate markers of the inflammatory response (Table 4). Plasma levels of CRP (7.9 ± 12.5 vs. 9.8 ± 11.9 mg/dL, *p*=0.018), but not of ferritin (675.0 ± 935.0 vs. 912.0 ± 1148.0 ng/mL, *p*=0.171) nor IL-6 (51.4 ± 82.6 vs. 54.0 ± 53.5 pg/mL, *p*=0.531), were significantly lower in the RASi group than in patients in the non-RASi group. When comparing the stop-RASi vs. non-stop-RASi groups, we found that only IL-6 levels differed, being significantly higher in those patients whose RASi drugs were stopped (88.6 ± 102.2 vs. 32.6 ± 50.0 pg/mL, *p*=0.024).

## 4. Discussion

Our main findings are in accordance with the current state of the art, in the sense that chronic treatment with RASi did not affect the severity of the disease and the risk of death during hospitalization for COVID-19. To the best of our knowledge, this is the first hospital-based study to enroll patients prospectively. In addition, this is also one of the few observational studies that specifically correlates the potential effect of RASi maintenance vs. withdrawing on clinical outcomes during hospitalization.

While the pandemic is rapidly evolving, the neutral effect of RASi exposure on mortality is increasingly recognized, although some disparities exist. Patoulias et al. [15] showed that, although the use of RASi does not increase the odds for SARS-CoV-2-related death in a global scenario (OR = 1.06, 95% CI 0.77–1.47, *I*^2^ = 83%), it increases the odds for death in Europe by 68% (OR = 1.68, 95% CI 1.05–2.70, *I*^2^ = 82%), while decreasing it in Asia by 38% (OR = 0.62, 95% CI 0.39–0.99, *I*^2^ = 0%). Others have come to the same nonsignificant results in a global perspective, but no subgroup analysis by region was made [16]. Our study indicates that RASi exposure is not associated with an increase nor decrease in the risk of inhospital all-cause mortality (adjusted OR 0.66, 95% CI 0.31–1.40, *p*=0.226), even though the odds showed a nonsignificant trend of decrease in the exposed group after adjusting for age and comorbidities (logistic regression coefficient of −0.42) (Table 2). These findings should contribute with new insights from a European country since most of the studies come from Asia where cardiovascular disease prevalence and its treatment strategies diverge greatly.

ARDS is the main cause of death in COVID-19 patients [7] and its prevalence is highly variable between studies, ranging from 15% to almost 33% [17]. In line with the literature, almost one-third of our patients developed ARDS/ALI. Decreased expression of ACE2 after viral infection can alter pulmonary vascular permeability leading to pulmonary oedema and acute respiratory failure [18]. This is believed to play a central role in SARS-CoV-2-related ARDS and justifies the theoretical protective role of RASi in this context. Unlike mortality, which is easily assessed and surely the most frequent outcome of interest in similar studies, illness severity criteria have been inconsistently defined across the studies addressing the impact of RASi in COVID-19 patients. In our study, we defined severe disease based on objective and easily accessible parameters, namely, the development of ARDS/ALI and the rates of ICU admission and IMV requirements. A recent meta-analysis [12] showed that only one study defined severe disease based on the development of ARDS. Unfortunately, the study was probably not powered enough to detect any difference between the two groups (RASi group, *n* = 31 vs. non-RASi group, *n* = 69; 0% vs. 13%, *p*=0.176), so no comparison can be inferred [19]. In the present study, we observed that the use of a RASi was not associated with the odds of developing ARDS/ALI, even after multivariable adjustment. These results should, however, be analysed with caution because it englobes not only ARDS patients diagnosed by Berlin criteria [13] but also patients that we classified as having ALI who would, otherwise, be classified as having ARDS should they be given a positive end-expiratory pressure. In our institution, this is only available in the ICU from where these subjects were not candidates due to their comorbid conditions to start with. We also observed no significant differences in the odds for ICU admission nor for IMV need between the two groups. We find these results expectable given that the development of ARDS/ALI is the main reason why these patients are admitted to an ICU and are ventilated.

We did notice that the median time from symptom onset to any of the secondary outcomes, but not to death, was significantly shorter in the RASi group than those in the non-RASi group. Giving that patients under RASi therapy presented sooner to the hospital, and assuming that medical care was equally given to both groups of patients (with a comparable median time from admission to each secondary outcome and a comparable proportion of inhospital treatments between the two groups (Table 1)), this suggests that patients in the RASi group may have been admitted with more severe disease.

The effect of inhospital withdrawing vs. maintaining the RASi prescription is poorly reported in the context of SARS-CoV-2 infection. Zhang et al. [20] showed that inpatient use of RASi was associated with a lower risk of 28-day all-cause inhospital mortality (adjusted hazard ratio 0.37; 95% CI, 0.15–0.89; *p*=0.03) than in RASi nonusers. Due to the small number of patients (*n* = 5) who started an ACEi or ARB upon admission, we could not analyse it separately. Our adjusted results show, however, that the withdrawing of RASi in the first three days of hospitalization was associated with a higher risk of developing ARDS/ALI but not of dying (Table 3). The opposite is necessarily true since patients were dichotomized in a binary way. These findings further support the guidance statements by several international societies on continuing current antihypertensive treatment during the COVID-19 pandemic [21]. Ongoing randomized clinical trials of RASi in COVID-19 patients (e.g., NCT04312009, NCT04366050, and NCT04355429) will certainly elucidate this in the near future.

A hyperinflammatory state characterizes the severe cases of COVID-19 [22]. Consistent with this hypothesis, levels of CRP, ferritin, IL-6, IL-8, and tumour necrosis factor alpha are significantly increased in deceased patients with COVID-19 compared to those who survived [23] and are independent predictors of survival [24]. The anti-inflammatory effect of ACEi/ARB use has been increasingly demonstrated in several preclinical and clinical disease models [25], but studies on COVID-19 are scarce [26]. Even though we did not aim to specifically capture the full inflammatory profile of SARS-CoV-2 infection, our study is one of the few observational studies to explore with some detail the effect of RASi exposure and inpatient management on inflammatory markers. We found that CRP levels on admission were significantly lower in patients receiving chronic RASi treatment, whereas no significant change was observed in IL-6 and ferritin levels. These findings have been inconsistently reported, with some authors showing similar [27, 28] and opposite [29] results. We also found that CRP and ferritin values did not differ significantly whether patients had maintained or discontinued their RASi treatment upon admission. Curiously, IL-6 levels did change, being significantly lower in those patients who maintained their RASi therapy regimen. A multicentric study regarding the effect of inhospital use of ACEi/ARB reported similar findings regarding CRP levels, but neither ferritin nor IL-6 was measured [20]. To study the correlation between the inflammatory markers and the measured outcomes was beyond the scope of the current study.

This study has several limitations. First, its single-centre and observational nature limits the generalisability and the establishment of causality. Second, we have excluded patients who, despite being tested positive for SARS-CoV-2 by RT-PCR, were admitted due to social reasons. We assume this could have driven our results towards the null as asymptomatic and, thus, less severe patients were less likely to have an adverse outcome. Third, when examining the effect of RASi withdrawing vs. its maintenance, we did not take into consideration whether patients were actually given the prescribed drug nor the effect of its introduction or suspension beyond the first three days of hospitalization. Finally, although more than half of the enrolled patients were followed prospectively and despite our active role in their inpatient treatment, we were not able to have direct interaction with all patients, neither did we have control over when or which laboratory exams were performed; hence, some data are missing. We also have some strengths. As all our patients ended the study, our mortality and severe outcome rate are accurate. In addition, the ambispective nature of the study, which is a unique feature in this kind of work, warrants preciseness in data collection.

## 5. Conclusion

In conclusion, chronic treatment with RASi is not associated with either inhospital mortality or disease severity as measured by the risk of developing ARDS/ALI, ICU admission, and need for IMV in COVID-19 patients. Our findings support the maintenance of RASi during hospitalization. Future nationwide and multinational studies should corroborate our results and address these pitfalls, particularly patients' enrollment criteria as well as severity outcome definitions, as this can help uniformize and generalize knowledge.

## Figures and Tables

**Table 1:** Baseline demographic, clinical, and laboratory characteristics, inpatient treatment, and outcome comparison in regard to RASi exposure.

	Total *n* = 279	RASi *n* = 133	Non-RASi *n* = 146	*p*
*Demographics*				
Male sex	159 (57)	77 (57.9)	82 (56.2)	0.771
Age, years	69 ± 26	76 ± 21	64 ± 30	**<0.001**
Caucasian ethnic	223/269 (82.9)	113/128 (88.3)	110/141 (78.0)	**0.026**

*Comorbid conditions*				
High blood pressure	185 (66.3)	127 (95.5)	58 (39.7)	**<0.001**
Dyslipidaemia	93 (33.3)	62 (46.6)	31 (21.2)	**<0.001**
Diabetes mellitus	76 (27.2)	53 (39.9)	23 (15.8)	**<0.001**
Cardiovascular disease^•^	74 (26.5)	48 (36.1)	26 (17.8)	**0.001**
Obesity^+^	71 (25.5)	43 (32.6)	28 (19.2)	**0.011**
Chronic kidney disease^♦^	51 (18.4)	31 (23.3)	20 (13.8)	**0.041**
Cerebrovascular disease°	49 (17.6)	24 (18.2)	25 (17.1)	0.817
Chronic obstructive pulmonary disease	37 (13.3)	21 (15.8)	16 (11)	0.235
Active cancer^⋄^	23 (8.2)	9 (6.8)	14 (9.6)	0.392
Asthma	15 (5.4)	8 (6.0)	7 (4.8)	0.652
Rheumatic/autoimmune disease^∴^	15 (5.4)	7 (5.3)	8 (5.5)	0.936
HIV/AIDS	5 (1.8)	1 (0.8)	4 (2.7)	0.211

*Other antihypertensive drugs*				
Diuretic	95 (34.1)	70 (52.6)	25 (17.1)	**<0.001**
Calcium channel blocker	77 (27.6)	56 (42.1)	21 (14.4)	**<0.001**
Beta-blocker	62 (22.3)	36 (27.1)	26 (17.8)	0.063

*Signs and symptoms*				
Fever ^*∗*^	206/277 (74.4)	97/132 (74.5)	109/145 (75.2)	0.748
Cough	185/276 (67.0)	77/131 (58.8)	108/145 (74.5)	**0.006**
Dyspnoea	166/277 (59.9)	82/132 (62.1)	84/145 (57.9)	0.477
Asthenia	146/275 (53.1)	65/130 (50.0)	81/145 (55.9)	0.331
Myalgia	84/273 (30.8)	35/130 (26.9)	49/143 (34.3)	0.189
Diarrhoea	64/276 (23.2)	31/131 (23.7)	33/145 (22.8)	0.859
Headache	40/273 (14.7)	10/130 (7.7)	30/143 (21)	**0.002**
Chest pain	37/273 (13.6)	18/130 (13.9)	19/143 (13.3)	0.893
Anorexia	33/273 (12.1)	13/130 (10.0)	20/143 (14)	0.313
Nausea/vomiting	32/276 (11.6)	15/131 (11.5)	17/145 (11.7)	0.943
Ageusia	21/271 (7.8)	5/129 (3.9)	16/142 (11.3)	**0.024**
Sore throat	18/272 (6.6)	8/129 (6.2)	10/143 (7)	0.813
Anosmia	17/271 (6.3)	4/129 (3.1)	13/142 (9.2)	**0.046**
Rhinorrhoea	8/273 (2.9)	4/129 (3.1)	4/144 (2.8)	1.000
Abdominal pain	8/274 (2.9)	2/130 (1.5)	6/144 (4.2)	0.287
Arthralgia	2/273 (0.7)	1/130 (0.8)	1/143 (0.7)	1.000

*Laboratory results*				
Hb, M: <13.0 g/dL/F: <12.0 g/dL	90/278 (32.4)	44/132 (33.3)	46 (31.5)	0.745
Leucocyte, >11000 × 10^6^/L	40/278 (14.4)	21/132 (15.9)	19 (13.0)	0.492
Neutrophil, >7500 × 10^6^/L	75/278 (27)	39/132 (29.6)	36 (24.7)	0.359
Lymphocyte, <1000 × 10^6^/L	126/276 (45.7)	65/132 (49.2)	61 (42.4)	0.252
500–1000 × 10^6^/L	103/276 (37.3)	56/132 (42.4)	47/144 (32.6)	0.093
≤500 × 10^6^/L	23/276 (8.3)	9/132 (6.8)	14/144 (9.7)	0.383
Absolute value, ×10^6^/L	1060 ± 600	1050 ± 630	1060 ± 560	0.761
Monocyte, >1000 × 10^6^/L	10/272 (3.7)	6/130 (4.6)	4/142 (2.8)	0.527
Platelet count, <150 × 10^9^/L	67/276 (24.3)	33/131 (25.2)	34/145 (23.5)	0.736
100–150 × 10^9^/L	58/276 (21.0)	30/131 (22.9)	28/145 (19.3)	0.465
≤100 × 10^9^/L	9/176 (3.3)	3/131 (2.3)	6/145 (4.1)	0.506
D-dimer, >0.25 *µ*g/mL	190/257 (73.9)	96/125 (76.8)	94/132 (71.2)	0.308
≤0.5 *µ*g/mL	78/257 (30.4)	40/125 (32.0)	38/132 (28.8)	0.576
0.5–1 *µ*g/mL	58/257 (22.6)	25/125 (20.0)	33/132 (25.0)	0.338
≥1 *µ*g/mL	54/257 (21.0)	31/125 (24.8)	23/132 (17.4)	0.147
Acute kidney injury ^*∗∗*^	94/269 (34.9)	54/127 (42.5)	40/142 (28.2)	**0.014**
Sodium, <135 mmol/L	85/275 (30.9)	47/132 (35.6)	38/143 (26.6)	0.105
AST, >40 U/L	103/274 (37.6)	47/130 (36.2)	56/144 (38.9)	0.641
ALT, >41 U/L	60/274 (21.9)	23/130 (17.7)	37/144 (25.7)	0.110
Total bilirubin, >1.2 mg/dL	9/242 (3.7)	6/115 (5.2)	3/127 (2.4)	0.315
Creatin kinase, >300 U/L	53/246 (21.5)	28/119 (23.5)	25/127 (19.7)	0.464
LDH, >250 U/L	223/272 (82)	100/130 (76.9)	123/142 (86.6)	**0.038**
Albumin, <3 g/dL	27/215 (12.6)	12/102 (11.8)	15/113 (13.3)	0.739
Troponin T, >14 ng/L	121/226 (53.5)	78/112 (69.6)	43/114 (37.7)	**<0.001**
CRP, >5 mg/dL	208/278 (74.8)	90/132 (68.2)	118 (80.8)	**0.015**
≤10 mg/dL	83/278 (29.9)	36/132 (27.3)	47 (32.2)	0.371
10–20 mg/dL	76/278 (27.3)	31/132 (23.5)	45 (30.8)	0.170
≥20 mg/dL	49/278 (17.6)	22/132 (16.7)	27 (18.5)	0.690
Absolute value, mg/dL	9.3 ± 12.4	7.9 ± 12.5	9.8 ± 11.9	**0.018**
Procalcitonin, >2 ng/mL	18/261 (6.9)	10/125 (8.0)	8/136 (5.9)	0.626
Ferritin, >300 ng/mL	158/183 (86.3)	71/85 (83.5)	87/98 (88.8)	0.303
300–1000 ng/mL	85/183 (46.5)	43/85 (50.6)	42/98 (42.9)	0.296
>1000 ng/mL	73/183 (39.9)	28/85 (32.9)	45/98 (45.9)	0.074
Absolute value, ng/mL	789.0 ± 1104.0	675.0 ± 935.0	912.0 ± 1148.0	0.171
IL-6, >40 pg/mL	46/76 (60.5)	16/29 (55.2)	30/47 (63.8)	0.453
Absolute value, pg/mL	54.0 ± 73.0	51.4 ± 82.6	54.0 ± 53.5	0.531

*Inpatient treatment*				
Hydroxychloroquine	143/271 (52.8)	74/128 (57.8)	69/143 (48.3)	0.116
Antiviral therapy (total)	193/270 (71.5)	93/127 (73.2)	100/143 (69.9)	0.549
Lopinavir/ritonavir	177/270 (65.6)	86/127 (67.7)	91/143 (63.6)	0.481
Remdesivir	21/270 (7.8)	9/127 (7.1)	12/143 (8.4)	0.689
Antibiotics	135/273 (49.5)	62/129 (48.1)	73/144 (50.7)	0.664
Tocilizumab	15/272 (5.5)	5/129 (3.9)	10/143 (7)	0.298
Steroids	78/269 (29.0)	35/127 (27.6)	43/142 (30.3)	0.623

*Timeframes* ^×^				
Time from disease onset to admission, days	5 ± 4	4 ± 4	6 ± 5	**0.041**
Time from disease onset to outcome, days	17 ± 12	17 ± 14	17 ± 12	0.792
Time from admission to outcome, days	10 ± 13	11 ± 13	10 ± 10.5	0.669

*Outcomes*				
Death	48/277 (17.3)	25/132 (18.9)	23/145 (15.9)	0.499
Time from disease onset to death, days	12 ± 22	12 ± 19	13 ± 22	0.732
Time from admission to death, days	7.5 ± 16	8 ± 14	7 ± 18	0.959
ARDS/ALI	87/269 (32.3)	43/128 (33.6)	44/141 (31.2)	0.676
Time from disease onset to ARDS, days	9 ± 7	7 ± 5	11 ± 6	**0.002**
Time from admission to ARDS, days	4 ± 5	3 ± 5	5 ± 5	0.166
ICU	89 (31.9)	44 (33.1)	45 (30.8)	0.686
Time from disease onset to ICU, days	8 ± 5	7 ± 5	8 ± 3.5	**0.044**
Time from admission to ICU, days	2 ± 3	1.5 ± 3	2 ± 1	0.939
Duration, days	12 ± 25	12 ± 23	14 ± 29	0.384
IMV	58 (20.8)	28 (21.1)	30 (20.6)	0.917
Time from disease onset to IMV, days	8 ± 5	6 ± 5	9 ± 4	**0.002**
Time from admission to IMV, days	2 ± 4	2 ± 4	3 ± 3	0.126
Duration, days	16 ± 21	12.5 ± 31	20 ± 19	0.593

Data are shown as number (%) for categorical variables and median ± interquartile range for continuous variables. The denominators of patients who were included in the analysis are provided if they differed from the overall numbers within the group. ^•^Cardiovascular disease included the following: aortic aneurysm disease, cardiomyopathy of any cause, coronary artery disease, heart failure of any cause, heart valve disease, peripheral artery disease, and pulmonary hypertension. ^+^Obesity was defined as body mass index equal to or higher than 30 kg/m^2^. ^♦^Chronic kidney disease was diagnosed according to the Kidney Disease: Improving Global Outcomes position statement. °Cerebrovascular disease included the following: ischemic and/or haemorrhagic stroke and cerebral microvascular disease. ^⋄^Cancer included any type of active solid and/or haematological cancer under active surveillance and/or treatment. ^∴^Rheumatic/autoimmune disease included the following: rheumatoid arthritis, systemic lupus erythematosus, and psoriatic arthritis  ^*∗*^Fever was defined as tympanic temperature of at least 38.0°C.  ^*∗∗*^Acute kidney injury was diagnosed according to Acute Kidney Injury Network criteria. ^×^The outcome date is the time the patient died or was clinically discharged (see Materials and Methods section). ALT, alanine aminotransferase; ARDS/ALI, acute respiratory distress syndrome/acute lung injury; AST, aspartate aminotransferase; CRP, c-reactive protein; F, female; Hb, haemoglobin; HIV/AIDS, human immunodeficiency virus/acquired immunodeficiency syndrome; ICU, intensive care unit; IL-6, interleukin 6; IMV, invasive mechanical ventilation; M, male; RASi, renin-angiotensin system inhibitors.

**Table 2:** Odds ratios for measured outcomes between RASi and non-RASi groups as a function of the patients' demographic characteristics and comorbid conditions.

	Crude model			Adjusted model		
	Coef.	OR (95% CI)	*p*	Coef.	OR (95% CI)	*p*
Death	0.21	1.24 (0.66–2.31)	0.500	- 0.42	0.66 (0.31–1.40)	0.279
ARDS/ALI	0.11	1.12 (0.67–1.86)	0.676	0.10	1.11 (0.66–1.87)	0.698
ICU admission	0.10	1.11 (0.67–1.84)	0.686	0.06	1.06 (0.63–1.79)	0.828
IMV need	0.03	1.03 (0.58–1.84)	0.917	0.01	1.01 (0.56–1.83)	0.962

ORs and *p* values were calculated with logistic regression analysis. Multivariable OR was adjusted as follows: death: age, high blood pressure, cardiovascular disease, and chronic obstructive pulmonary disease; ARDS/ALI: sex; ICU admission: sex; and chronic obstructive pulmonary disease and IMV: sex. ARDS/ALI, acute respiratory distress syndrome/acute lung injury; CI, confidence interval; Coef., logistic regression coefficient; ICU, intensive care unit; IMV, invasive mechanical ventilation; OR, odds ratio; RASi, renin-angiotensin system inhibitors.

**Table 3:** Odds ratios for primary and secondary outcomes among RASi patients who stopped their RASi drugs during hospitalization.

	Crude model			Adjusted model		
	Coef.	OR (95% CI)	*p*	Coef.	OR (95% CI)	*p*
Death	1.48	4.38 (1.11–17.21)	**0.035**	1.54	4.67 (0.87–24.98)	0.072
ARDS/ALI development	1.47	4.33 (1.49–12.59)	**0.007**	1.51	4.54 (1.53–13.44)	**0.006**
ICU admission	0.7	2.00 (0.73–5.47)	0.175	0.81	2.24 (0.79–6.39)	0.131
MV need	0.82	2.27 (0.53–9.72)	0.269	0.95	2.58 (0.58–11.55)	0.216

ORs and *p* values were calculated with logistic regression analysis. Multivariable OR was adjusted for age for death and for sex for ARDS/ALI, ICU admission, and IMV need. ARDS/ALI, acute respiratory distress syndrome/acute lung injury; CI, confidence interval; Coef., logistic regression coefficient; ICU, intensive care unit; IMV, invasive mechanical ventilation; OR, odds ratio; RASi, renin-angiotensin system inhibitors.

**Table 4:** Comparison of the inflammatory markers' levels between RASi/non-RASi and stop-RASi/non-stop-RASi groups.

	RASi	Non-RASi	*p*	Stop-RASi	Non-stop-RASi	*p*
CRP, mg/dL	7.9 ± 12.5	9.8 ± 11.9	**0.018**	8.4 ± 12.7	6.3 ± 8.1	0.473
Ferritin, ng/mL	675.0 ± 935.0	912.0 ± 1148.0	0.171	693.0 ± 906.0	604.0 ± 724.5	0.169
IL-6, pg/mL	51.4 ± 82.6	54.0 ± 53.5	0.531	88.6 ± 102.2	32.6 ± 50.0	**0.024**

Data are shown as median ± interquartile range. CRP, C-reactive protein; IL-6, interleukin 6.

## Data Availability

The collected data used to support the findings of this study are restricted by the Ethics Committee of Hospital de Santa Maria in order to protect patient privacy. Data are available for researchers who meet the criteria for access to confidential data.

## Supplementary Materials

Supplementary Materials Supplementary Table 1: baseline demographic and clinical characteristics and inpatient treatment comparison according to primary outcome. Supplementary Table 2: baseline demographic and clinical characteristics and inpatient treatment comparison according to ARDS/ALI development. Supplementary Table 3: baseline demographic and clinical characteristics and inpatient treatment comparison according to ICU admission. Supplementary Table 4: baseline demographic and clinical characteristics and inpatient treatment comparison according to IMV requirement. Supplementary Table 5: baseline demographic and clinical characteristics, inpatient treatment, and outcome comparison in regard to inpatient RASi management.
