# Supplementary material for: The Effect of Chronic and Inhospital Exposure to Renin-Angiotensin System Inhibitors on the Outcome and Inflammatory State of Coronavirus Disease 2019 Adult Inpatients
Source: Int J Hypertens. 2021 Mar 8;2021:5517441. doi: 10.1155/2021/5517441 (PMC7985238; doi:10.1155/2021/5517441)
Supplement: Supplementary Materials — Supplementary Table 1: baseline demographic and clinical characteristics and inpatient treatment comparison according to primary outcome. Supplementary Table 2: baseline demographic and clinical characteristics and inpatient treatment comparison according to ARDS/ALI development. Supplementary Table 3: baseline demographic and clinical characteristics and inpatient treatment comparison according to ICU admission. Supplementary Table 4: baseline demographic and clinical characteristics and inpatient treatment comparison according to IMV requirement. Supplementary Table 5: baseline demographic and clinical characteristics, inpatient treatment, and outcome comparison in regard to inpatient RASi management. [file 5517441.f1.docx]

**Supplementary Material**

[Gaspar P et al. *The Effect of Chronic and In-hospital Exposure to Renin-Angiotensin System Inhibitors on the Outcome and Inflammatory State of Coronavirus Disease 2019 Adult Inpatients*. Int J Hypertens, 2021]

**Supplementary Table 1.** Baseline demographic and clinical characteristics and inpatient treatment comparison according to primary outcome.

| **Variable** | **Total**  n = 277 | **Non-survivor**  n = 48 | **Survivor**  n = 229 | ***p*** |
| --- | --- | --- | --- | --- |
| ***Demographics*** |  |  |  |  |
| Male sex | 158 (57.0) | 28 (58.3) | 130 (56.8) | 0.842 |
| Age, years | 69 ± 26 | 84.5 ± 16.5 | 67 ± 25 | <0.001 |
| Caucasian ethnic | 222/267 (83.2) | 41/45 (91.1) | 181/222 (81.5) | 0.118 |
| ***Comorbid conditions*** |  |  |  |  |
| High blood pressure | 184 (66.4) | 40 (83.3) | 144 (62.9) | 0.006 |
| Diabetes mellitus | 75 (27.1) | 16 (33.3) | 59 (25.8) | 0.283 |
| Dyslipidaemia | 91 (32.9) | 16 (33.3) | 75 (32.6) | 0.938 |
| Obesity | 69 (24.9) | 6 (12.5) | 63 (27.5) | 0.029 |
| Cardiovascular disease | 74 (26.7) | 24 (50.0) | 50 (21.8) | <0.001 |
| Cerebrovascular disease | 49 (17.7) | 14 (29.2) | 35 (15.3) | 0.022 |
| Chronic kidney disease | 51 (18.5) | 15 (31.3) | 36 (15.8) | 0.012 |
| Chronic obstructive pulmonary disease | 37 (13.4) | 14 (29.2) | 23 (10.0) | <0.001 |
| Cancer | 23 (8.3) | 6 (12.5) | 17 (7.4) | 0.246 |
| Asthma | 15 (5.4) | 1 (2.1) | 14 (6.1) | 0.481 |
| Rheumatic/autoimmune disease | 15 (5.4) | 0 | 15 (6.6) | NA |
| HIV/AIDS | 4 (1.4) | 0 | 4 (1.8) | NA |
| ***Chronic medication*** |  |  |  |  |
| RASi | 132 (47.7) | 25 (52.1) | 107 (46.7) | 0.499 |
| ACEi | 66 (50.0) | 13 (52.0) | 53 (49.5) | 0.824 |
| ARB | 66 (50.0) | 12 (48.0) | 54 (50.5) | 0.824 |
| Diuretic | 95 (34.3) | 25 (52.1) | 70 (30.6) | 0.004 |
| Calcium channel blocker | 77 (27.8) | 17 (35.4) | 60 (26.2) | 0.195 |
| Beta-blocker | 62 (22.4) | 16 (33.3) | 46 (20.1) | 0.045 |
| ***Inpatient medication*** |  |  |  |  |
| RASi management |  |  |  |  |
| RASi initiated | 5/103 (4.9) | 1/16 (6.3) | 4/87 (4.6) | 0.578 |
| RASi stopped | 42/87 (48.3) | 10/13 (76.9) | 32/74 (43.2) | 0.025 |
| RASi maintained | 45/87 (51.8) | 3/13 (23.1) | 42(74 (56.8) | 0.025 |
| Hydroxychloroquine | 141/269 (52.4) | 26/42 (61.9) | 115/229 (50.7) | 0.180 |
| Anti-viral therapy (total) | 191/268 (71.3) | 25/42 (59.5) | 166/226 (73.5) | 0.067 |
| Lopinavir/ritonavir | 175/268 (66.3) | 25/42 (59.5) | 150/226 (66.4) | 0.392 |
| Remdesivir | 21/268 (7.8) | 0 | 21/226 (9.3) | NA |
| Antibiotics | 133/271 (49.1) | 30/43 (69.8) | 103/228 (45.2) | 0.003 |
| Tocilizumab | 14/270 (5.2) | 1/43 (2.3) | 13/227 (5.7) | 0.705 |
| Steroids | 77/267 (28.8) | 10/39 (25.6) | 67/228 (29.4) | 0.633 |
| ***Timeframe*** |  |  |  |  |
| Time from disease onset to admission, days | 5 (4) | 3 (4) | 6 (4) | 0.008 |

Data is shown as number (%) for categorical variables and median ± interquartile range for continuous variables. The denominators of patients who were included in the analysis are provided if they differed from the overall numbers within the group. ACEi, angiotensin-converting enzyme inhibitor; ARB, angiotensin II receptor blocker; ARDS/ALI, acute respiratory distress syndrome/acute lung injury; HIV/AIDS, human immunodeficiency virus/acquired immunodeficiency syndrome; ICU, intensive care unit; IMV, invasive mechanical ventilation; RASi, renin-angiotensin system inhibitor.

Note: see the main manuscript’s **Table 1** legend to a detailed specification of comorbid conditions.

**Supplementary Table 2**. Baseline demographic and clinical characteristics and inpatient treatment comparison according to ARDS/ALI development.

| **Variable** | **Total**  n = 269 | **ARDS/ALI**  n = 87 | **No ARDS/ALI**  n = 182 | ***p*** |
| --- | --- | --- | --- | --- |
| ***Demographics*** |  |  |  |  |
| Male sex | 156 (58) | 63 (72.4) | 93 (51.1) | 0.001 |
| Age, years | 69 ± 26 | 68 ± 22 | 69.5 ± 28 | 0.644 |
| Caucasian ethnic | 216/260 (83.1) | 69/82 (84.2) | 147/178 (82.6) | 0.813 |
| ***Comorbid conditions*** |  |  |  |  |
| High blood pressure | 176 (65.4) | 54 (62.1) | 122 (67.0) | 0.423 |
| Diabetes mellitus | 73 (27.1) | 25 (28.7) | 48 (26.4) | 0.684 |
| Dyslipidaemia | 90 (33.5) | 30 (34.5) | 60 (33) | 0.805 |
| Obesity | 70 (26.0) | 26 (29.9) | 44 (24.2) | 0.318 |
| Cardiovascular disease | 70 (26.0) | 21 (24.1) | 49 (26.9) | 0.626 |
| Cerebrovascular disease | 47 (17.5) | 13 (14.9) | 34 (18.7) | 0.450 |
| Chronic kidney disease | 50 (18.6) | 14 (16.1) | 36 (19.8) | 0.467 |
| Chronic obstructive pulmonary disease | 35 (13.0) | 15 (17.2) | 20 (11) | 0.154 |
| Cancer | 22 (8.2) | 5 (5.8) | 17 (9.3) | 0.314 |
| Asthma | 15 (5.4) | 4 (4.6) | 11 (6.0) | 0.780 |
| Rheumatic/autoimmune disease | 14 (5.2) | 3 (3.5) | 11 (7.0) | 0.559 |
| HIV/AIDS | 5 (1.9) | 1 (1.2) | 4 (2.2) | 1.000 |
| ***Chronic medication*** |  |  |  |  |
| RASi | 128 (47.6) | 43 (49.4) | 85 (46.7) | 0.676 |
| ACEi | 63 (49.2) | 20 (46.5) | 43 (50.6) | 0.663 |
| ARB | 65 (50.8) | 23 (53.5) | 42 (49.4) | 0.663 |
| Diuretic | 91 (33.8) | 28 (32.2) | 63 (34.6) | 0.693 |
| Calcium channel blocker | 73 (27.1) | 24 (27.6) | 49 (26.9) | 0.909 |
| Beta-blockers | 59 (21.9) | 17 (19.5) | 42 (23.1) | 0.512 |
| ***Inpatient medication*** |  |  |  |  |
| RASi management |  |  |  |  |
| RASi initiated | 5/100 (5.0) | 0 | 5/71 (7.0) | NA |
| RASi stopped | 40/85 (47.1) | 16/22 (72.7) | 24/63 (38.1) | 0.005 |
| RASi maintained | 45/85 (52.9) | 6/22 (27.3) | 39/63 (61.9) | 0.005 |
| Hydroxychloroquine | 138/263 (52.5) | 50/81 (61.7) | 88 (48.4) | 0.045 |
| Anti-viral therapy | 187/262 (71.4) | 62/80 (77.5) | 125 (68.7) | 0.146 |
| Lopinavir/ritonavir | 171/262 (65.3) | 57/80 (71.3) | 114 (62.6) | 0.177 |
| Remdesivir | 21/262 (8.0) | 8/80 (10.0) | 13 (7.1) | 0.433 |
| Antibiotics | 130/265 (49.1) | 57/83 (68.7) | 73 (40.1) | <0.001 |
| Tocilizumab | 14/264 (5.3) | 9/82 (11) | 5 (2.8) | 0.014 |
| Steroids | 76/261 (29.1) | 38/80 (47.5) | 38/181 (21) | <0.001 |

Data is shown as number (%) for categorical variables and median ± interquartile range for continuous variables. The denominators of patients who were included in the analysis are provided if they differed from the overall numbers within the group. ACEi, angiotensin-converting enzyme inhibitor; ARB, angiotensin II receptor blocker; ARDS/ALI, acute respiratory distress syndrome/acute lung injury; HIV/AIDS, human immunodeficiency virus/acquired immunodeficiency syndrome; ICU, intensive care unit; IMV, invasive mechanical ventilation; RASi, renin-angiotensin system inhibitor.

Note: see the main manuscript’s **Table 1** legend to a detailed specification of comorbid conditions.

**Supplementary Table 3.** Baseline demographic and clinical characteristics and inpatient treatment comparison according to ICU admission.

| **Variable** | **Total**  n = 279 | **ICU**  n = 89 | **No ICU**  n = 190 | ***p*** |
| --- | --- | --- | --- | --- |
| ***Demographics*** |  |  |  |  |
| Male sex | 159 (57) | 67 (75.3) | 92 (48.4) | <0.001 |
| Age, years | 69 ± 26 | 67 ± 21 | 72 ± 27 | 0.051 |
| Caucasian ethnic | 223/269 (82.9) | 67/84 (79.8) | 156/185 (84.3) | 0.512 |
| ***Comorbid conditions*** |  |  |  |  |
| High blood pressure | 185 (66.3) | 55 (61.8) | 130 (68.4) | 0.275 |
| Diabetes mellitus | 76 (27.2) | 25 (28.1) | 51 (26.8) | 0.827 |
| Dyslipidaemia | 93 (33.3) | 32 (36) | 61 (32.1) | 0.525 |
| Obesity | 71 (25.5) | 29 (32.6) | 42 (22.1) | 0.061 |
| Cardiovascular disease | 74 (26.5) | 20 (22.5) | 54 (28.4) | 0.294 |
| Cerebrovascular disease | 49 (17.6) | 9 (10.1) | 40 (21.1) | 0.025 |
| Chronic kidney disease | 51 (18.3) | 13 (14.6) | 38 (20.0) | 0.277 |
| Chronic obstructive pulmonary disease | 37 (13.3) | 18 (20.2) | 19 (10.0) | 0.019 |
| Cancer | 23 (8.2) | 4 (4.5) | 19 (10.0) | 0.119 |
| Asthma | 15 (5.4) | 4 (4.5) | 11 (5.8) | 0.781 |
| Rheumatic/autoimmune disease | 15 (5.4) | 2 (2.3) | 13 (6.8) | 0.156 |
| HIV/AIDS | 5 (1.8) | 1 (1.1) | 4 (2.1) | 1.000 |
| ***Chronic medication*** |  |  |  |  |
| RASi | 133 (47.7) | 44 (49.4) | 89 (46.8) | 0.686 |
| ACEi | 67 (50.4) | 20 (45.5) | 47 (52.8) | 0.425 |
| ARB | 66 (49.6) | 24 (54.6) | 42 (47.2) | 0.425 |
| Diuretic | 95 (34.1) | 30 (33.7) | 65 (34.2) | 0.934 |
| Calcium channel blocker | 77 (27.6) | 28 (31.5) | 49 (25.8) | 0.323 |
| Beta-blockers | 62 (22.2) | 21 (23.6) | 41 (21.6) | 0.706 |
| ***Inpatient medication*** |  |  |  |  |
| RASi management |  |  |  |  |
| RASi initiated | 5/104 (4.8) | 2/31 (6.5) | 3/73 (4.1) | 0.633 |
| RASi stopped | 43/88 (48.9) | 13/21 (61.9) | 30/67 (44.8) | 0.171 |
| RASi maintained | 45/88 (51.1) | 8/21 (38.1) | 37/67 (55.2) | 0.171 |
| Hydroxychloroquine | 143/271 (52.8) | 48/81 (59.3) | 95 (50.0) | 0.162 |
| Anti-viral therapy | 193/270 (71.5) | 66/80 (82.5) | 127 (66.8) | 0.009 |
| Lopinavir/ritonavir | 177/270 (66.3) | 60/80 (75.0) | 117 (61.6) | 0.034 |
| Remdesivir | 21/270 (7.8) | 7/80 (8.8) | 14 (7.4) | 0.699 |
| Antibiotics | 135/273 (49.5) | 53/83 (63.9) | 82 (43.2) | 0.002 |
| Tocilizumab | 15/272 (5.5) | 9/82 (11) | 6 (3.2) | 0.010 |
| Steroids | 78/269 (29.0) | 38/79 (48.1) | 40 (21.1) | <0.001 |

Data is shown as number (%) for categorical variables and median ± interquartile range for continuous variables. The denominators of patients who were included in the analysis are provided if they differed from the overall numbers within the group. ACEi, angiotensin-converting enzyme inhibitor; ARB, angiotensin II receptor blocker; ARDS/ALI, acute respiratory distress syndrome/acute lung injury; HIV/AIDS, human immunodeficiency virus/acquired immunodeficiency syndrome; ICU, intensive care unit; IMV, invasive mechanical ventilation; RASi, renin-angiotensin system inhibitor.

Note: see the main manuscript’s **Table 1** legend to a detailed specification of comorbid conditions.

**Supplementary Table 4.** Baseline demographic and clinical characteristics and inpatient treatment comparison according to IMV requirement.

| **Variable** | **Total**  n = 279 | **IMV**  n = 58 | **No IMV**  n = 221 | ***p*** |
| --- | --- | --- | --- | --- |
| ***Demographics*** |  |  |  |  |
| Male sex | 159 (57) | 44 (75.9) | 115 (52.0) | 0.001 |
| Age, years | 69 ± 26 | 64.5 ± 18 | 72 ± 28 | 0.059 |
| Caucasian ethnic | 223/269 (82.9) | 45/55 (81.8) | 178/214 (83.2) | 0.654 |
| ***Comorbid conditions*** |  |  |  |  |
| High blood pressure | 185 (66.3) | 34 (58.6) | 151 (68.3) | 0.164 |
| Diabetes mellitus | 76 (27.2) | 15 (25.9) | 61 (27.6) | 0.791 |
| Dyslipidaemia | 93 (33.3) | 19 (32.8) | 74 (33.5) | 0.917 |
| Obesity | 71 (25.5) | 15 (25.9) | 56 (25.3) | 0.935 |
| Cardiovascular disease | 74 (26.5) | 11 (19) | 63 (28.5) | 0.143 |
| Cerebrovascular disease | 49 (17.6) | 7 (12.1) | 42 (19.0) | 0.217 |
| Chronic kidney disease | 51 (18.3) | 8 (13.8) | 43 (19.5) | 0.321 |
| Chronic obstructive pulmonary disease | 37 (13.3) | 12 (20.7) | 25 (11.3) | 0.061 |
| Cancer | 23 (8.2) | 3 (5.2) | 20 (9.1) | 0.339 |
| Asthma | 15 (5.4) | 2 (3.5) | 13 (5.9) | 0.744 |
| Rheumatic/autoimmune disease | 15 (5.4) | 2 (3.5) | 13 (5.9) | 0.744 |
| HIV/AIDS | 5 (1.8) | 1 (1.7) | 4 (1.8) | 1.000 |
| ***Chronic medication*** |  |  |  |  |
| RASi | 133 (47.7) | 28 (48.3) | 105 (47.5) | 0.917 |
| ACEi | 67 (50.4) | 14 (50.0) | 53 (50.5) | 0.964 |
| ARB | 66 (49.6) | 14 (50.0) | 52 (49.5) | 0.964 |
| Diuretic | 95 (34.1) | 16 (27.6) | 79 (35.8) | 0.243 |
| Calcium channel blocker | 77 (27.6) | 16 (27.6) | 61 (27.6) | 0.998 |
| Beta-blockers | 62 (22.2) | 12 (20.7) | 50 (22.6) | 0.752 |
| ***Inpatient medication*** |  |  |  |  |
| RASi management |  |  |  |  |
| RASi initiated | 5/104 (4.8) | 0 | 5/86 (5.8) | NA |
| RASi stopped | 43/88 (48.9) | 6/9 (66.7) | 37/79 (46.8) | 0.259 |
| RASi maintained | 45/88 (51.1) | 3/9 (33.3) | 42/79 (53.2) | 0.259 |
| Hydroxychloroquine | 143/271 (52.8) | 36/51 (70.6) | 107/220 (48.6) | 0.005 |
| Anti-viral therapy | 193/270 (71.5) | 43/50 (86.0) | 150/220 (68.2) | 0.012 |
| Lopinavir/ritonavir | 177/270 (65.6) | 43/50 (86.0) | 134/220 (60.9) | 0.001 |
| Remdesivir | 21/270 (7.8) | 1/50 (2.0) | 20/220 (9.1) | 0.091 |
| Antibiotics | 135/273 (49.5) | 135/53 (66.0) | 100/220 (45.5) | 0.007 |
| Tocilizumab | 15/272 (5.5) | 8/52 (15.4) | 7/220 (3.2) | 0.002 |
| Steroids | 78/269 (29.0) | 25/50 (50.0) | 53/219 (24.2) | <0.001 |

Data is shown as number (%) for categorical variables and median ± interquartile range for continuous variables. The denominators of patients who were included in the analysis are provided if they differed from the overall numbers within the group. ACEi, angiotensin-converting enzyme inhibitor; ARB, angiotensin II receptor blocker; ARDS/ALI, acute respiratory distress syndrome/acute lung injury; HIV/AIDS, human immunodeficiency virus/acquired immunodeficiency syndrome; ICU, intensive care unit; IMV, invasive mechanical ventilation; RASi, renin-angiotensin system inhibitor.

Note: see the main manuscript’s **Table 1** legend to a detailed specification of comorbid conditions.

**Supplementary Table 5.** Baseline demographic and clinical characteristics, inpatient treatment and outcome comparison in regard to inpatient RASi management.

| **Variable** | **Total**  n = 88 | **Stopped RASi**  n = 43 | **Non-stop-RASi**  n = 45 | ***p*** |
| --- | --- | --- | --- | --- |
| ***Demographics*** |  |  |  |  |
| Male sex | 47 (53.4) | 22 (51.2) | 25 (55.6) | 0.680 |
| Age, years | 76 ± 21 | 80 ± 16 | 71 ± 24 | 0.033 |
| Caucasian ethnic | 72/85 (84.7) | 36/40 (90.0) | 36 (80.0) | 0.045 |
| ***Comorbid conditions*** |  |  |  |  |
| High blood pressure | 84 (95.5) | 42 (97.7) | 42 (93.3) | 0.328 |
| Diabetes mellitus type 2 | 36 (40.9) | 18 (41.9) | 18 (40.0) | 0.859 |
| Dyslipidaemia | 40 (45.5) | 17 (39.5) | 23 (51.1) | 0.276 |
| Obesity | 31 (35.2) | 11 (25.6) | 20 (44.4) | 0.064 |
| Cardiovascular disease | 35 (39.8) | 18 (41.9) | 17 (37.8) | 0.696 |
| Cerebrovascular disease | 16 (18.1) | 8 (18.6) | 8 (17.8) | 1.000 |
| Chronic kidney disease | 19 (21.6) | 9 (20.9) | 10 (22.2) | 1.000 |
| Chronic obstructive pulmonary disease | 12 (13.6) | 5 (11.6) | 7 (15.6) | 0.758 |
| Asthma | 6 (6.8) | 2 (4.7) | 4 (8.9) | 0.677 |
| Cancer | 6 (6.8) | 3 (7) | 3 (6.7) | 1.000 |
| Rheumatic/autoimmune disease | 5 (5.7) | 2 (4.7) | 3 (6.7) | 1.000 |
| HIV/AIDS | 1 (1.1) | 1 (2.3) | 0 | NA |
| Chronic medication |  |  |  |  |
| Beta-blockers | 28 (31.8) | 14 (32.6) | 14 (31.1) | 0.884 |
| Calcium channel blocker | 38 (43.2) | 20 (46.5) | 18 (40.0) | 0.538 |
| Diuretic | 45 (51.1) | 26 (60.5) | 19 (42.2) | 0.087 |
| ***Inpatient treatment*** |  |  |  |  |
| Hydroxychloroquine | 43/87 (49.4) | 19/42 (45.2) | 24 (53.3) | 0.450 |
| Anti-viral therapy (total) | 61/87 (70.1) | 28/42 (66.7) | 33 (73.3) | 0.497 |
| Lopinavir/ritonavir | 55/87 (63.2) | 25/42 (59.5) | 30 (66.7) | 0.490 |
| Remdesivir | 8/87 (9.2) | 4/42 (9.5) | 4 (8.9) | 1.000 |
| Antibiotics | 39 (44.3) | 25 (58.1) | 14 (31.1) | 0.011 |
| Tocilizumab | 4 (4.6) | 4 (9.3) | 0 | NA |
| Steroids | 26/87 (29.9) | 14/42 (33.3) | 12 (26.7) | 0.497 |
| ***Outcomes*** |  |  |  |  |
| Death | 13/87 (14.9) | 10/42 (23.8) | 3 (6.7) | 0.035 |
| Time from disease onset to death, days | 12 ± 19 | 8.5 ± 6 | 6 ± 4 | 0.106 |
| Time from admission to death, days | 8 ± 14 | 7 ± 5 | 5 ± 5 | 0.105 |
| ARDS/ALI | 22 (25.9) | 16 (40.0) | 6 (13.3) | 0.005 |
| Time from disease onset to ARDS, days | 7 ± 5 | 6 ± 4 | 8 ± 4 | 0.299 |
| Time from admission to ARDS, days | 3 ± 5 | 3 ± 3 | 3 ± 4 | 0.473 |
| ICU | 21 (23.9) | 13 (30.2) | 8 (17.8) | 0.171 |
| Time from disease onset to ICU, days | 7 ± 5 | 8 ± 6 | 8 ± 6.5 | 0.846 |
| Time from admission to ICU, days | 1.5 ± 3 | 2 ± 3 | 1.5 ± 2 | 0.319 |
| Duration, days | 12 ± 23 | 11 ± 14 | 6.5 ± 11.5 | 0.384 |
| IMV | 9 (10.2) | 6 (14) | 3 (6.7) | 0.309 |
| Time from disease onset to IMV, days | 6 ± 5 | 7 ± 8 | 11 ± 9 | 0.763 |
| Time from admission to IMV, days | 2 ± 4 | 3.5 ± 2 | 4 ± 5 | 0.685 |
| Duration, days | 12.5 ± 31 | 23.5 ± 48.5 | 10 ± 27 | 0.858 |

Data is shown as number (%) for categorical variables and median ± interquartile range for continuous variables. The denominators of patients who were included in the analysis are provided if they differed from the overall numbers within the group. ACEi, angiotensin-converting enzyme inhibitor; ARB, angiotensin II receptor blocker; ARDS/ALI, acute respiratory distress syndrome/acute lung injury; HIV/AIDS, human immunodeficiency virus/acquired immunodeficiency syndrome; ICU, intensive care unit; IMV, invasive mechanical ventilation; RASi, renin-angiotensin system inhibitor.

Note: see the main manuscript’s **Table 1** legend to a detailed specification of comorbid conditions.
